# A Draft Mitogenome of *Plectus Murrayi*

**DOI:** 10.2478/jofnem-2022-0035

**Published:** 2022-09-30

**Authors:** Xia Xue, Byron J. Adams, Adler R. Dilman

**Affiliations:** 1 Henan Key Laboratory of Helicobacter pylori & Microbiota and Gastrointestinal Cancer, Marshall Medical Research Center, The Fifth Affiliated Hospital of Zhengzhou University, Henan, China; 2 Department of Biology and Evolutionary Ecology Laboratories, Brigham Young University (BYU), Provo, UT, USA; 3 BYU Life Science Museum, Provo, UT, USA; 4 Department of Nematology, University of California, Riverside, CA, USA

**Keywords:** Antarctica, genomics, genome decay, mitochondrial genome, MitoZ, phylogeny

## Abstract

*Plectus murrayi* is a free-living microbivorous nematode endemic to Antarctic soils. Our draft assembly of its mitogenome was 15,656 bp long, containing 12 protein-coding, eight transfer RNA (tRNA), and two ribosomal RNA (rRNA) genes. Mitophylogenomic analyses extend our understanding of mitochondrial evolution in Nematoda

## Announcement

*Plectus murrayi* is a free-living, microbivorous, limnoterrestrial nematode endemic to the ice-free Antarctic soils (Andrassy, 1998). The Plectidae are of particular interest for resolving patterns and processes of nematode evolution because they are the sister taxon to the Rhabditida (Blaxter et al., 1998; Holterman et al., 2006; Blaxter and Koutsovoulos, 2015). Moreover, *P. murrayi’s* high tolerance to environmental stresses (desiccation, freezing, high concentration of heavy metals, etc.) makes it a good model for discovering the limits of life and understanding mechanisms of extreme stress survival, including cryptobiosis (Nkem et al., 2005; Adhikari et al., 2009, 2010; Adhikari and Adams, 2011; Wharton and Raymond, 2015).

The genome of *P. murrayi* was extracted from a population of approximately 5,000 nematodes and sequenced using an Illumina 2000 Genome Analyzer IIx sequencer in a paired-end mode, as described previously (Xue et al., 2021). The mitogenome was assembled and annotated using MitoZ (V2.4) based on the genomic raw reads. The pipeline includes filtering, de novo assembly, HMMER (Hidden Markov Models) and protein-coding gene (PCG) annotation, and visualization using Circos (Meng et al., 2019). The resulting mitochondrial genome assembly was 15,652 bp in length. Twelve PCGs, two ribosomal RNA (rRNA) genes (r-rRNA and s-rRNA), and eight transfer RNA (tRNA) genes were identified in this mitogenome (Fig. S1 in Supplementary Material). The overall nucleotide composition was 26.80% A, 21.58% C, 21.55% G, and 25.01% T, and the G+C content was 45.30%. Most of the 12 PCGs used ATN as the start codon (ATT for COX1, COX2, COX3, ND1, ATP6, and CYTB; ATA for ND2, ND4, and ND5). ND4L, ND3, and ND5 began with the codon TTG. The stop codon TAA was assigned to most of the PCGs (COX2, ND1, ATP6, ND2, ND4, ND6, COX1, CYTB, ND5, and COX3), but an incomplete stop codon was used by two PCGs (ND3, and ND4L).

**Figure S1:**
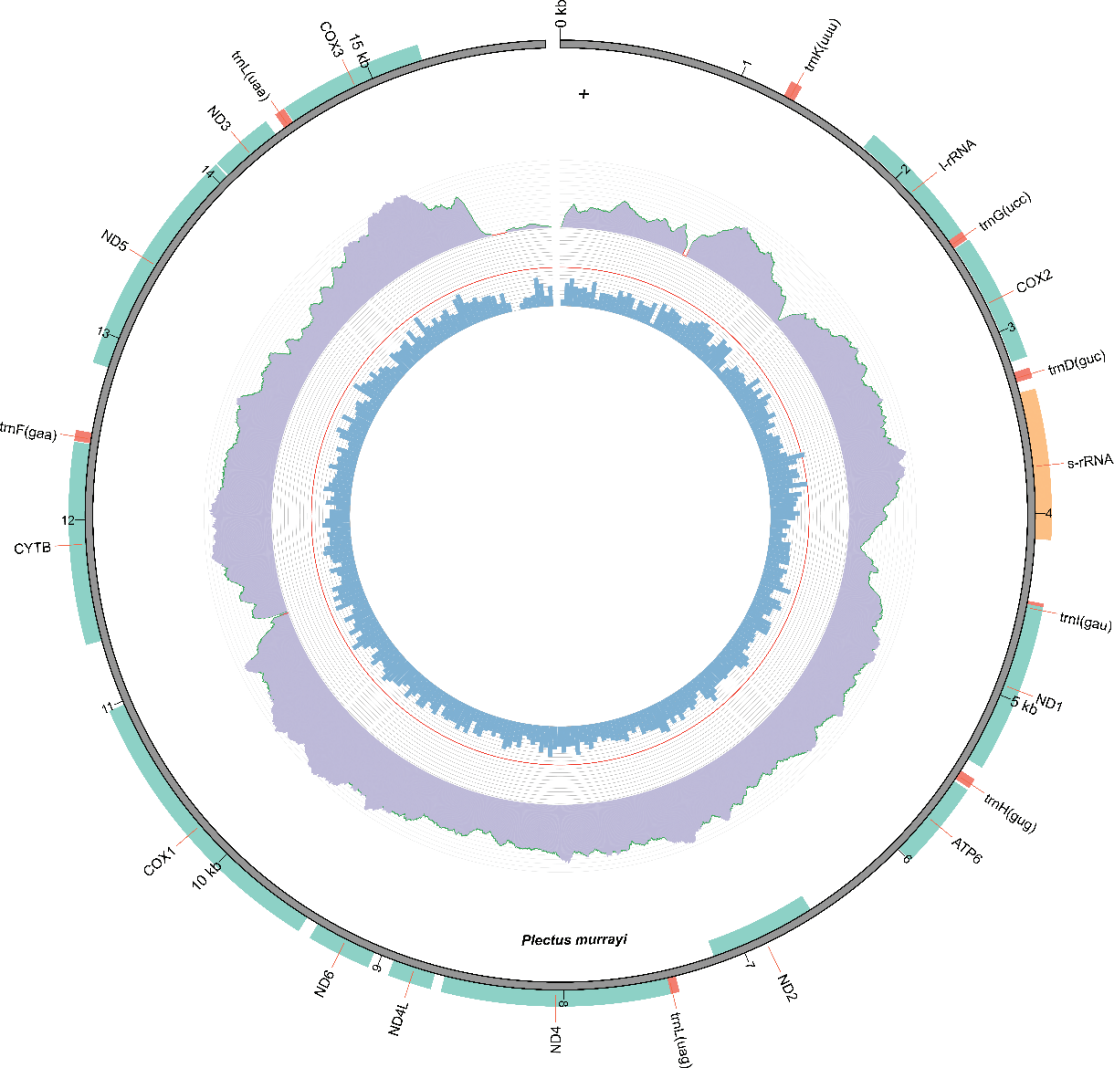
The Circos draft mitogenome of *Plectus murrayi* assembled based on MitoZ. The inner and outside of the circos refer to different directions of this draft mitogenome on which all genes were mapped.

Mitochondrial genomes of other nematodes available from GenBank were used to perform mitophylogenomic analysis. A maximum-likelihood analysis using IQ-Tree software (Minh et al., 2020) based on a matrix of aligned PCGs, tRNAs, and rRNAs by Clustal (Sievers et al., 2011) confirms a monophyletic Plectidae as sister clade to the Rhabditida, with *P. murrayi* as sister to *P. acuminatus* and *P. aquatilis*.

This study provides an example of assembling and annotating nematode mitogenomes based on existing genomic data (Meng et al., 2019). It is notable that we recovered fewer tRNA genes than expected, based on existing assemblies of *P. aquatilis* and *P. acuminatus* (Kim et al., 2017). However, we note the absence of ATP8 (Supplementary Fig. S1), which is also missing from other nematode mitogenomes (Kim et al., 2017)_._ Loss of these genes could be an artifact of our analyses, but it is consistent with findings from the *P. murrayi* genome, which suggests that such losses by way of genome decay are an adaptive response to the harsh Antarctic environment (Xue et al., 2021). Our work shows that mining and assembling mitogenomes from whole-genome data can be a powerful tool for understanding the evolution of mitochondria in Nematoda and, in particular, understanding adaptive variations of functional genes related to energy generation and allocation.

## Data submission

Nucleotide accession numbers associated with this announcement are PRJNA317772 (BioProject) and SAMN04625768 (SAMN04625768) which are openly available in GenBank.
